# Towards mechanisms-driven strategy for persistent atrial fibrillation ablation: Leveraging digital twins

**DOI:** 10.1016/j.premed.2026.100036

**Published:** 2026-03-28

**Authors:** Kensuke Sakata, Natalia A. Trayanova

**Affiliations:** a Alliance for Cardiovascular Diagnostic and Treatment Innovation, Johns Hopkins University, 3400 N. Charles St. Hackerman Hall 213, Baltimore, MD, 21218, USA; b Department of Biomedical Engineering, Johns Hopkins University, 3400 N. Charles St. Wyman Park Building Suite 400 West, Baltimore, MD, 21218, USA

**Keywords:** Persistent atrial fibrillation, Substrate ablation, Digital twin, Driver, Fibrosis

## Abstract

Atrial fibrillation (AF) is the most common sustained arrhythmia, affecting 1–2% of the global population, and is a major cause of stroke and heart failure. With the population aging, its prevalence is expected to increase further, imposing a growing burden on healthcare systems. However, the gold standard treatment—pulmonary vein isolation with catheter ablation that primarily prevents pulmonary vein triggers from initiating fibrillatory conduction—has limited efficacy in the persistent form of AF (PsAF), which is characterized by atrial fibrotic remodeling. Thus far, numerous intra-atrial electrogram-based mapping approaches have been developed to identify PsAF arrhythmogenic substrate locations capable of attracting reentries (LRs) and guide substrate modification in clinical practice; however, their clinical effectiveness remains controversial and the optimal ablation strategy remains unclear. Furthermore, extensive substrate ablation may adversely affect patients post-ablation due to scar-related atrial tachycardia or impaired atrial function despite successful AF control. Recently, personalized heart digital twins (DTs) have emerged as a promising technology for precision medicine, enabling non-invasive patient-specific reconstruction of cardiac electrical activity using clinical imaging and electrophysiological data. Personalized DTs allow investigation of patient-specific electrophysiological behavior, prediction of arrhythmia inducibility, and identification of arrhythmogenic substrates pre-procedurally. In this review, we summarize the mechanisms underlying PsAF maintenance, current limitations of PsAF ablation therapy, and recent advances in DT technology, highlighting its potential to facilitate mechanism-driven, personalized ablation planning and improve PsAF patient care.

## 1. Introduction

Atrial fibrillation (AF) is the most common cardiac arrhythmia; an estimated 59.7 million people worldwide had AF/atrial flutter as of 2019, nearly a twofold rise compared to 1990 (Roth et al., 2020). Given that age-adjusted incidence and prevalence rates have remained stable, the growing burden of AF may be owing to an ageing population exposed to increasing comorbidities (Karatela & Calkins, 2025). Adverse outcomes associated with AF include its increased risk of stroke, dementia, myocardial infarction, sudden cardiac death, heart failure, chronic kidney disease, and peripheral arterial disease (Joglar et al., 2024).

Catheter ablation as an initial therapy is superior to drug therapy (Andrade et al., 2021) and pulmonary vein (PV) isolation (PVI), mainly preventing PV triggers from initiating fibrillatory conduction, is the cornerstone therapy for AF (Haïssaguerre et al., 2000). However, compared with paroxysmal AF (Kuniss et al., 2021; Wazni et al., 2021), its success rate for the persistent form of AF (PsAF) remains suboptimal with approximately 50 to 60% (Kistler et al., 2023; Verma et al., 2015). This limited success for PsAF patients typically stems from the arrhythmogenic substrate with fibrosis progression and atrial remodeling (Platonov et al., 2011), driving sustaining reentries (sometimes called rotors) which underlie AF (Pandit & Jalife, 2013; Yamamoto & Trayanova, 2022). Accordingly, over the last two decades, various strategies for PsAF extra-PVI ablation targeting rotational activities assumed to contain AF drivers (rotors) and/or abnormal substrate has been proposed (Calvo et al., 2017; Haissaguerre et al., 2014; Hirokami et al., 2023; Kumagai et al., 2013; Marrouche et al., 2022; Nademanee et al., 2004; Nakagawa et al., 2009; Narayan et al., 2012; Rolf et al., 2014; Sakata et al., 2018; Seitz et al., 2017); however, optimal targets and their detection methods remains unclear.

Recently, heart digital twins (DTs), a novel technology increasingly being used in precision medicine and cardiology, have emerged as a promising tool to enable a personalized approach to disease and health prevention, diagnosis, treatment, and management (Khoshfekr Rudsari et al., 2025; Niederer et al., 2019; Tortora et al., 2025; Yin et al., 2024). By combining mechanistic and statistical models, DTs leverage both population and individual representations to improve clinical decision-making despite practical limitations in clinical data acquisition (Corral-Acero et al., 2020). Here, population-level representation refers to statistical models informed by large-scale population data, which are integrated with patient-specific mechanistic models. One of the most advanced applications of heart DTs is in cardiac electrophysiology, where they enable investigation of electrophysiological mechanisms spanning multiple spatial scales, from cellular to the organ level, that cannot be directly evaluated in clinical procedures (Horrach et al., 2025). By integrating imaging and electrophysiological data, DTs allow simulation of patient-specific electrophysiological behavior, testing of arrhythmia induction, or identification of patient-specific arrhythmogenesis (Bhagirath et al., 2024; Trayanova & Prakosa, 2024). DT advances were made possible by significant developments in multiscale mechanistic computational modeling of the heart, and the atria specifically, as outlined in our previous review publications (Heijman et al., 2021; Trayanova, 2014; Trayanova et al., 2024).

This review article examines the achievements in atrial digital twins that could be leveraged towards the development of a mechanisms-driven strategy for PsAF ablation. Specifically, we first summarize the current landscape of PsAF treatment, and its clinical problems. Next, we outline the potential utility of atrial DTs in addressing these clinical issues, with a focus on their use in uncovering mechanistic insights into PsAF and exploring optimal ablation strategies, thereby facilitating personalized PsAF treatment and patient care.

## 2. PsAF arrhythmogenic substrate

The pathophysiological mechanism of AF persistence and progression is rooted in both atrial electrophysiological and structural remodeling (Allessie et al., 2002), led by inflammation associated with many systemic diseases such as coronary artery disease, hypertension, and obesity (Hu et al., 2015). In addition, inflammation promoted by AF itself perpetuates AF—called “AF begets AF” (Wijffels et al., 1995). Electrophysiological remodeling of atrial cardiomyocytes involves alterations in ion channel expression and calcium handling, which shorten the atrial action potential duration and refractory period, thereby reducing wavelength and facilitating the maintenance of reentrant activity (Allessie et al., 2002; Vagos et al., 2018). Remodeling of calcium channels and intracellular calcium cycling proteins also attenuates cytosolic calcium changes, resulting in impaired contractile function and atrial dilatation. This leads to atrial stretch, which promotes fibrotic and connexin remodeling. At the cellular level, fibroblast proliferation and differentiation into myofibroblasts promote structural remodeling through enhanced collagen deposition (Souders et al., 2009; Vasquez et al., 2010), and induce electrophysiological alterations via gap junction remodeling, characterized by altered connexin expression and distribution (Kato et al., 2012). Early studies employing personalized atrial models showed the formation of arrhythmogenic substrate accompanied by electrical slow conduction and block following fibroblasts proliferation (Ashihara et al., 2012; McDowell et al., 2013), and indeed, PsAF patients have been reported to exhibit greater degree of fibrosis than paroxysmal AF (Platonov et al., 2011).

Within such an electro-structural remodeled substrate, two major mechanistic hypotheses have been proposed to explain the maintenance of AF: the multiple wavelet hypothesis and the rotor-driver hypothesis.

According to the multiple wavelet hypothesis, AF is maintained by multiple wavelets randomly propagating throughout both atria along continuously changing pathways, primarily determined by tissue refractory periods (Moe et al., 1964). Subsequently, mapping in canine atria demonstrated that the simultaneous presence of multiple independent wavelets is necessary to perpetuate AF (Allessie et al., 1985). Later, mapping human atria provided the first direct evidence of such multiple-wavelet behavior (Konings et al., 1994). Further extending this concept, their group proposed that the co-existence of complex multiple-wavelets macroreentrant circuits, facilitated by fibrosis-related longitudinal dissociation of atrial muscle bundle and endocardial–epicardial dissociation, is a key for PsAF maintenance (Allessie et al., 2010; Verheule et al., 2010).

The Jalife group demonstrated spiral wave activity, providing the electrophysiological basis for the rotor-driver hypotheses in AF (Davidenko et al., 1992; Pertsov et al., 1993). Subsequently, optical mapping demonstrated that organized rotational activity can be observed during fibrillation (Gray et al., 1998). They further described a hierarchical organization of reentrant activity, with dominant rotors giving rise to daughter wavelets—the mother-rotor concept (Jalife et al., 2002; Mandapati et al., 2000; Samie et al., 2001). In addition, human mapping showed that rotors can meander, producing rapid and irregular electrical activity (Ikeda et al., 1997).

## 3. Substrate ablation

Understanding that AF persistence is driven by complex electro-structural remodeling has provided the rationale for substrate ablation strategies aimed at targeting arrhythmogenic tissue responsible for AF maintenance. Since PVI aiming at trigger elimination was developed (Haïssaguerre et al., 2000), various strategies for substrate modulation have been explored. Electrogram-based ablation targeting complex fractionated atrial electrograms (CFAEs) was proposed as indirect indicators of PsAF arrhythmogenesis (Nademanee et al., 2004). CFAEs are characterized by fractionation with multiple deflections or continuous baseline perturbation and/or very short cycle lengths. Later, CFAE-guided defragmentation within the left atrium (LA) and right atrium (RA) was integrated with line ablation at the LA roof and mitral isthmus, termed stepwise ablation, targeting AF termination during ablation (Haïssaguerre et al., 2005). They showed 95% freedom from atrial tachyarrhythmias (ATAs) at 11 months after multiple procedures, with mechanical function restoration despite a high incidence of post-ablation atrial tachycardia (AT) (40%) due to extensive ablation at the first session. Further, dominant frequency analysis of local electrograms integrating CFAE analysis was reported to be effective in both acute- and long-term ablation outcomes (Kumagai et al., 2013). Thus, CFAE ablation became a mainstream approach in the PsAF treatment.

However, subsequent randomized control trials showed disappointing outcomes of these strategies in succession: The STAR AF II reported that both additional CFAE ablation and linear ablation resulted in no improvement in AF freedom compared to PVI alone (Verma et al., 2015); the CHASE-AF showed the stepwise approach did not provide additional benefit over PVI alone, despite significant longer procedural and fluoroscopic duration (Vogler et al., 2015); and the EARNEST-PVI did not show the efficacy of CFAE or line ablation added to PVI (Inoue et al., 2021).

As an alternative to AF mapping-based strategies, ablation directly targeting low-voltage area (LVA), assumed to represent abnormal atrial substrate potentially underlying PsAF arrhythmogenesis, was proposed (Rolf et al., 2014). This approach was based on multiple previous clinical studies showing that, when applying a bipolar voltage threshold of 0.5 mV, LVAs were associated with atrial remodeled substrate (Kistler et al., 2004; Roberts-Thomson et al., 2009; Sanders et al., 2004; Sanders et al., 2003). However, most recently, the SUPPRESS-AF demonstrated in a multicenter randomized controlled trial that LVA ablation added to PVI did not improve 1-year ATA freedom in PsAF patients with LA LVAs (61% vs. 50%) (Masuda et al., 2025). Moreover, based on the insights using surgical biopsy into the relationship between atrial fibrotic remodeling and late gadolinium enhancement magnetic resonance imaging (LGE-MRI) signal intensity (McGann et al., 2014), targeting LA fibrosis detected by LGE-MRI was proposed in the DECAAF II; however, this approach was also comparable to PVI alone (43% vs. 46% at 1 year) (Marrouche et al., 2022). Importantly, despite the fact that atrial remodeling is not limited to the LA, these substrate ablation strategies targeted LVAs and fibrosis only within the LA, which may be associated with suboptimal outcomes.

**Fig. 1.**
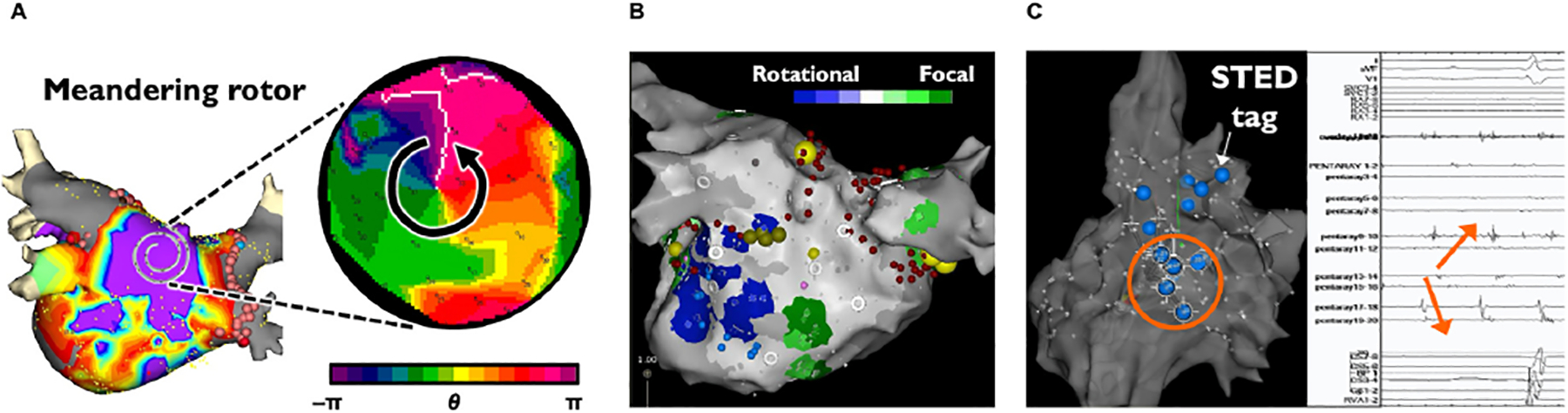
Recent approaches to driver identification during atrial fibrillation mapping A. ExTRa mapping B. CARTOFINDER C. Spatiotemporal electrogram dispersion (STED).

Furthermore, visualization-based AF mapping approaches have emerged in the past decade. An offline temporal phase mapping system using a 64-pole basket catheter was developed and reported to be useful for successful ablation targeting rotors and focal impulse sources, which were identified in almost all patients (Narayan et al., 2012). However, a recent randomized controlled trial, the REAFFIRM, has described that ablation targeting these AF-sustaining sources had no additional benefit over the PVI-only strategy (69% vs. 68% at 1 year) (Brachmann et al., 2025). This may be attributable to relatively coarse spatial resolution of basket-catheter-mapping, considering the size of rotor cores observed in animal experiments (Takahashi, 2024). In addition, an electrocardiographic imaging (ECGI) system, developed by the Rudy group (Ghosh et al., 2008), was utilized to map AF by recording body-surface potentials using a 252-electrode vest (Haissaguerre et al., 2014); however, the AFACART reported that, while ECGI-guided driver ablation resulted in 1-year freedom rate from AF of 77%, 49% of them had AT recurrence, which meant that only 39% of the entire cohort was free from ATAs (Knecht et al., 2017). This might be explained by the following limitations: ECGI-based rotor identification is fundamentally limited by the inverse problem, as many atrial phase singularities do not project onto the body surface (Rodrigo et al., 2014), and moreover, phase--mapping–based analyses applied to ECGI signals is prone to generating false rotors under clinically relevant conditions (Vijayakumar et al., 2016). More recently, the ExTRa Mapping (Nihon Kohden Corporation), a real-time online mapping tool, has been introduced as a promising approach for targeting potential AF driver areas (Sakata et al., 2018) (Fig. 1A). In this system, using a 20-pole electrode catheter, 41 bipolar signals (32 physical and 9 virtual) are used to generate phase maps based on simulated action potentials, allowing assessment of both wavefront propagation and wavefront–wavetail interaction. Recent clinical studies have reported improved freedom from ATAs compared with PVI alone (the ROTATE; 85% vs. 68% at 1 year) (Kawaji et al., 2025) and with LA posterior wall isolation plus PVI (77% vs. 45% at 3 years) (Okano et al., 2025). However, this system is currently available only in Japan, and therefore, further multicenter trials would be desirable.

Meanwhile, a few novel electrogram-based ablation strategies have been proposed for effective driver modulation. The CARTOFINDER system (Biosense Webster, Inc.) was first clinically validated for AF driver identification and its effective control, which detects focal and/or rotational activations by analyzing 30-s unipolar signals (Calvo et al., 2017) (Fig. 1B). However, the presence of AF drivers identified by CARTOFINDER post-PVI did not necessarily underlie AF recurrence (Takahashi et al., 2020), and therefore, future randomized controlled trials are warranted. Another strategy targeting spatiotemporal electrogram dispersion (STED) areas was reported (Seitz et al., 2017) (Fig. 1C). This approach detected potential rotor locations where rotational activities were observed, by identifying clusters of electrograms with spatiotemporal dispersion across ≥3 adjacent bipoles over the AF cycle length. A recent multicenter randomized control trial, the TAILORED-AF, demonstrated that ablation guided by artificial intelligence-detection of STED resulted in favorable outcomes in terms of 1-year freedom from AF compared to PVI-only (88% vs. 70%) (Deisenhofer et al., 2025). However, it should be noted that by including AT recurrence, their outcomes exhibited no difference between two strategies (76% vs. 71%). In this context, the potential for re-emergence of an era of AF mapping–guided strategies aiming at AF termination has attracted a considerable attention. Representative strategies for PsAF substrate ablation are summarized in Table 1.

## 4. Holistic patient-centered PsAF management

It is intuitive to expect that replacing all atrial tissue with (non-conductive) scar tissue would indeed abolish ATAs—but at what cost? As discussed in the preceding section, extra-PVI ablation, including linear ablation (Masuda et al., 2023), CFAE ablation (Lim et al., 2024), LVA ablation (Masuda et al., 2025), spatiotemporal electrogram dispersion ablation (Sakata et al., 2023; Deisenhofer et al., 2025), and surgical ablation (Takahashi et al., 2016), have frequently been associated with post-ablation ATs, which are often challenging to treat due to the difficulty in identifying their underlying mechanisms. When AF termination or AF freedom is primarily evaluated as the ablation efficacy endpoint, post-ablation AT needs to be “100%” treatable; otherwise, event-freedom from any ATAs would be the more appropriate clinical outcome measure. Given that some patients may develop more severe symptoms following PsAF ablation than prior to the PsAF ablation, requiring frequent cardioversion, continued strong antiarrhythmic drugs, or even pacemaker implantation due to uncontrollable ATs, special caution should be exercised with AT recurrence from the perspective of holistic patient-centered arrhythmia management. In addition, extensive ablation increases the risk of atrial stiffening and impaired atrial diastolic function (Khurram et al., 2016; Lee et al., 2021; Park et al., 2019).

AF is associated with adverse prognosis, yet it remains uncertain to what extent restoration of sinus rhythm, as a binary outcome of AF recurrence, improves hard clinical outcomes (Packer et al., 2019). Recently, it is emphasized that the perspective of holistic patient-centered arrhythmia management, i.e., hard clinical endpoints, is important, rather than “a strictly electrophysiological perspective” (Boriani et al., 2025). Traditionally, ablation efficacy has been evaluated focusing on softer surrogate markers, e.g., arrhythmia recurrences. Many studies assessing outcomes beyond binary ATA recurrence have emerged. While catheter ablation significantly reduced AF recurrence compared with drug therapies, it did not reduce all-cause mortality (Packer et al., 2019). Likewise, the impact of PsAF on long-term outcomes, i.e., all-cause mortality or all-cause hospitalization, is primarily attributable to medical comorbidities, rather than arrhythmia substrate or the PsAF diagnosis itself (Cheung et al., 2025). In heart failure patients with AF (mainly PsAF), binary AF recurrence after ablation was not associated with improved mortality or reduced heart failure hospitalization, but associated with lower AF burden (Brachmann et al., 2021). Similarly, healthcare utilization after ablation was not determined by binary recurrence outcome, but by AF recurrence type (paroxysmal AF or PsAF) (Crowley et al., 2024). Notably, a clear discordance between patient-centered treatment goals and traditional electrophysiological end points showed that most patients prioritize reduction in AF frequency over whether complete freedom from ATA events would be achieved or not (Zenger et al., 2024).

Given the current clinical dilemma of achieving AF control without extensive ablation, based on the historical experience of substrate ablation, these findings on the assessment of outcomes beyond binary ATA recurrence suggest that sustained efforts are needed to implement a more cautious strategy that integrates appropriate stratification of patients who would benefit from additional ablation to PVI and selective approaches narrowing ablation targets. At least, in the current era, where rapid technological advances have begun to enable large-scale data processing and the use of non-invasive tools for precise medicine, it is suggested that an ad-hoc strategy may no longer represent optimal patient care.

## 5. DTs for addressing clinical issues of PsAF ablation

**Fig. 2.**
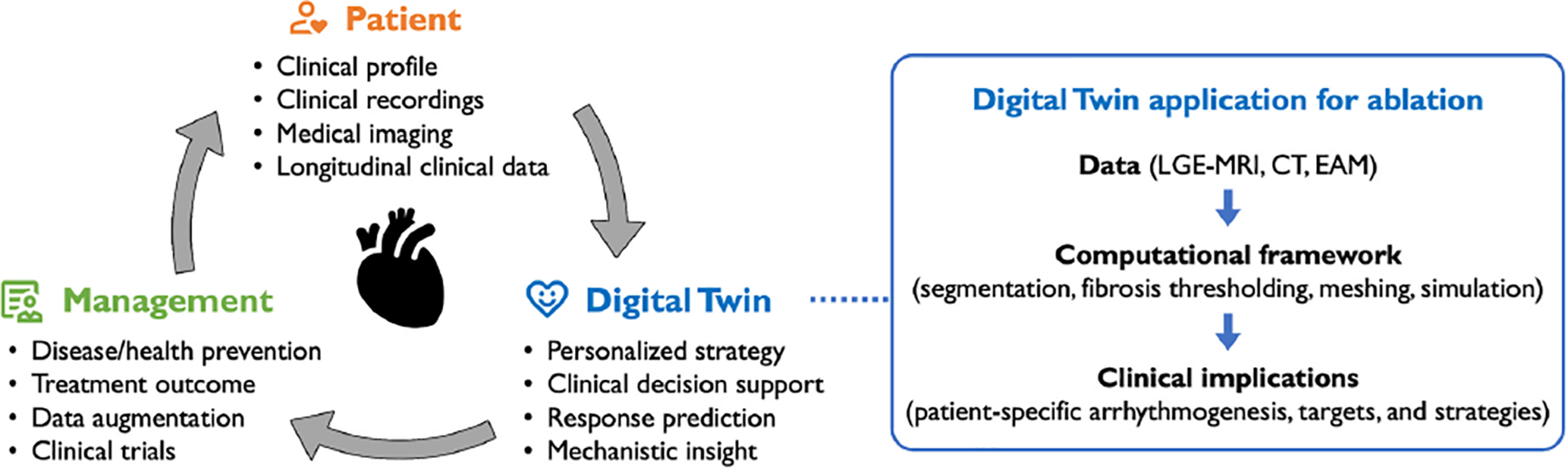
Clinical workflow of digital twin application CT = computed tomography; EAM = electroanatomic map; LGE-MRI = late gadolinium enhancement magnetic resonance imaging.

As the field moves toward selective, mechanism-based, and patient-centered arrhythmia management strategies, precise identification of AF drivers (rotors) becomes increasingly essential. However, current intracardiac bipolar rotor mapping techniques may have inherent limitations in accurately identifying AF drivers; Rodrigo et al. demonstrated that bipolar electrograms are subject to signal distortion arising from the forward problem, resulting in impaired phase reconstruction and potential misidentification of rotational activity (Rodrigo et al., 2017). Recently, there has been growing interest in non-invasive, mechanism-based approaches that do not rely solely on instantaneous electrical measurements. One such emerging concept is personalized heart DTs, which integrate patient-specific anatomy, electrophysiology, and tissue properties to virtually simulate arrhythmia behavior. Fig. 2 illustrates the clinical workflow of heart DT application, demonstrating its potential role in facilitating clinical decision-making in disease diagnosis and management and in guiding personalized therapeutic strategies.

In the DT framework, a central principle thus far has been the exclusive use of clinical data obtained before the ablation procedure—often non-invasive, e.g., LGE-MRI visualizing the distribution of structural remodeling such as fibrosis—combined with cell- and tissue-scale electrophysiological models representing human atrial myocardium, the latter obtained from biophysical experiments often enhanced with retrospective intra-procedural electrical recordings (Corrado et al., 2018; Krummen et al., 2012).

**Fig. 3.**
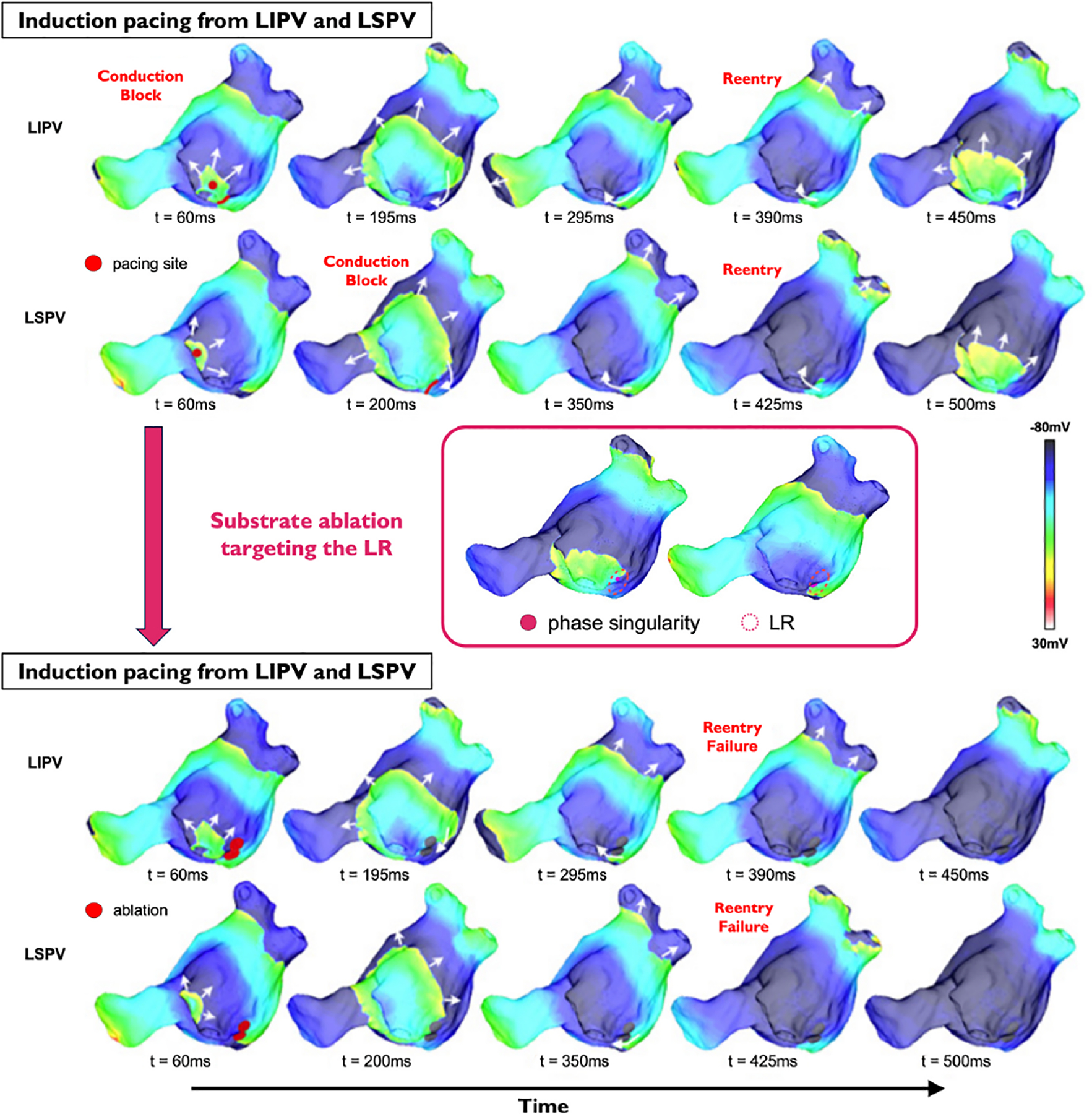
*In silico* substrate ablation targeting LRs (top) AF initiation in substrates from different pacing sites at LIPV (upper row) and LSPV (lower row). White arrows indicate direction of propagation. (middle) substrate ablation targeting an LR. Pink dot indicates the phase singularity location and red circular dashed line indicates the outline of the confined region of the persistent phase singularities. (bottom) LR ablation achieved AF non-inducibility. Red dot indicates ablation lesions. AF = atrial fibrillation; LIPV = left inferior pulmonary vein; LR = location of reentry; LSPV = left superior pulmonary vein. Adapted from McDowell et al., *PloS one*, 2016.

A histological validation study supported the association between increased LGE signal intensity and atrial fibrosis (McGann et al., 2014). Based on this foundation, personalized LGE-MRI-derived DTs incorporating the fibrosis-remodeled atria have been utilized to address clinical limitation of PsAF. Personalized DTs identified arrhythmogenic substrate, i.e., a potential Location capable of sustaining a Reentry (LR), which drives complex atrial activities consisting of many wavefront breakthroughs, unsustained reentries, and apparent disorganization, and showed that these activities can be terminated by substrate ablation targeting LRs (McDowell et al., 2015) (Fig. 3).

Furthermore, bi-atrial DTs revealed that the high degree of spatial interdigitation in the fibrosis distribution plays an important role in the formation of LRs, considered to be extra-PVI ablation targets in PsAF (Sakata, Bradley, Prakosa, Yamamoto, Ali, et al., 2024; Zahid et al., 2016). In addition to fibrosis patterns, patient-specific atrial DTs provided critical insights into the structural determinants of LRs, demonstrating that LRs preferentially localize to regions of reduced atrial wall thickness, thereby highlighting structural features that may serve as potential ablation targets (Kulathilaka et al., 2025).

Meanwhile, LR prediction based on “non-invasively and pre-procedurally” obtainable clinical data was reported to be sensitive to patient-specific tissue/cell-scale EP abnormalities (Deng et al., 2017). However, a comprehensive pacing protocol covering the entire atrial walls enabled uncovering of all LRs independently of changes in electrophysiological properties, even though the order in which the LRs were revealed varied depending on those properties (Hakim et al., 2018). This study highlights that specific fibrosis distribution exhibits an “anchoring” capacity at specific locations—the LRs, largely unrelated to patient-specific electrophysiological properties.

Additionally, the spatial resolution of tetrahedral finite-element discretization derived from LGE-MRI can influence LR prediction; however, its robustness has been validated (Boyle et al., 2021). This study compared meshes with edge lengths of ≈350 μm and ≈400 μm and found no differences in LR distribution or arrhythmia phenotype. Mesh refinement only produced transient conduction slowing without introducing false conduction pathways or new LRs, supporting the suitability for stable simulation results while faithfully preserving MRI-defined geometry and fibrosis.

**Fig. 4.**
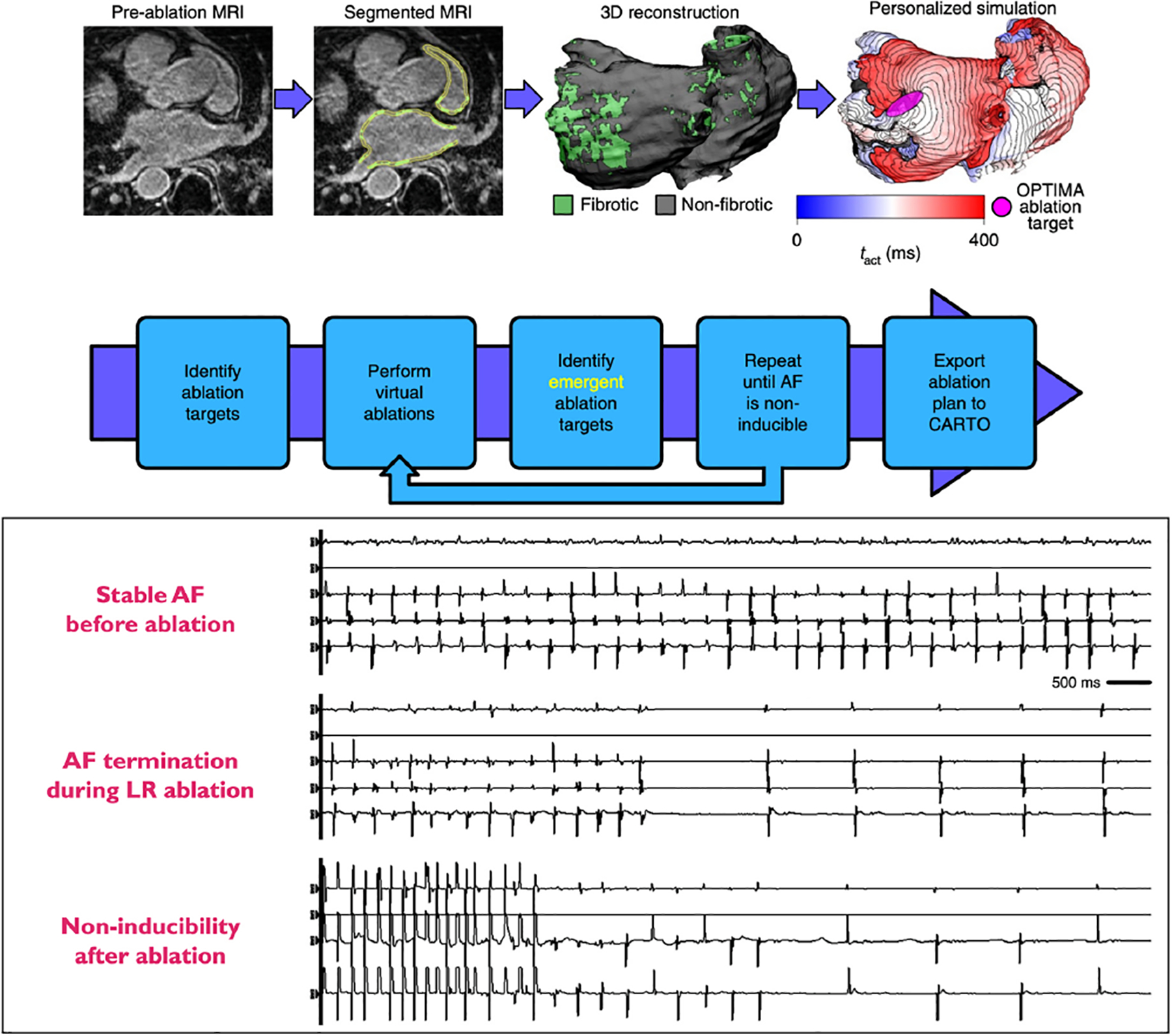
Flowchart of clinical application of DT-based ablation (top) Bi-atrial DTs incorporating fibrosis distribution were constructed from MRI images. Following LR identification by burst pacing at 40 bi-atrial sites, LRs were virtually ablated. Testing arrhythmia non-inducibility after each LR ablation and ablating any newly emerging LRs were repeated until no arrhythmia was inducible in the DT. The final set of DT-derived lesions was imported into the CARTO system and targeted during the ablation procedure. (bottom) Sustained AF observed before ablation was terminated during LR ablation, and non-inducibility of any arrhythmia was confirmed after ablation. AF = atrial fibrillation; DT = digital twin; LR = location of reentry; MRI = magnetic resonance imaging; OPTIMA = optimal target identification via modeling of arrhythmogenesis approach. Adapted from Boyle et al., *Nature biomedical engineering*, 2019.

As a clinical application of DTs to PsAF ablation, a pilot prospective study of ten patients with PsAF, the ablation of whom was driven by the DT predictions, demonstrated that ablating targets predicted from personalized DTs was associated with reduced AF recurrence—only one patient underwent redo ablation for atrial flutter without AF recurrence (Boyle et al., 2019) (Fig. 4). In this study, once LRs were identified by burst pacing from 40 bi-atrial sites, LRs were virtually ablated (all-LRs ablation strategy). Testing arrhythmia non-inducibility after each LR ablation and ablating any newly emerging LRs were repeated until no arrhythmia was inducible in the DT.

**Fig. 5.**
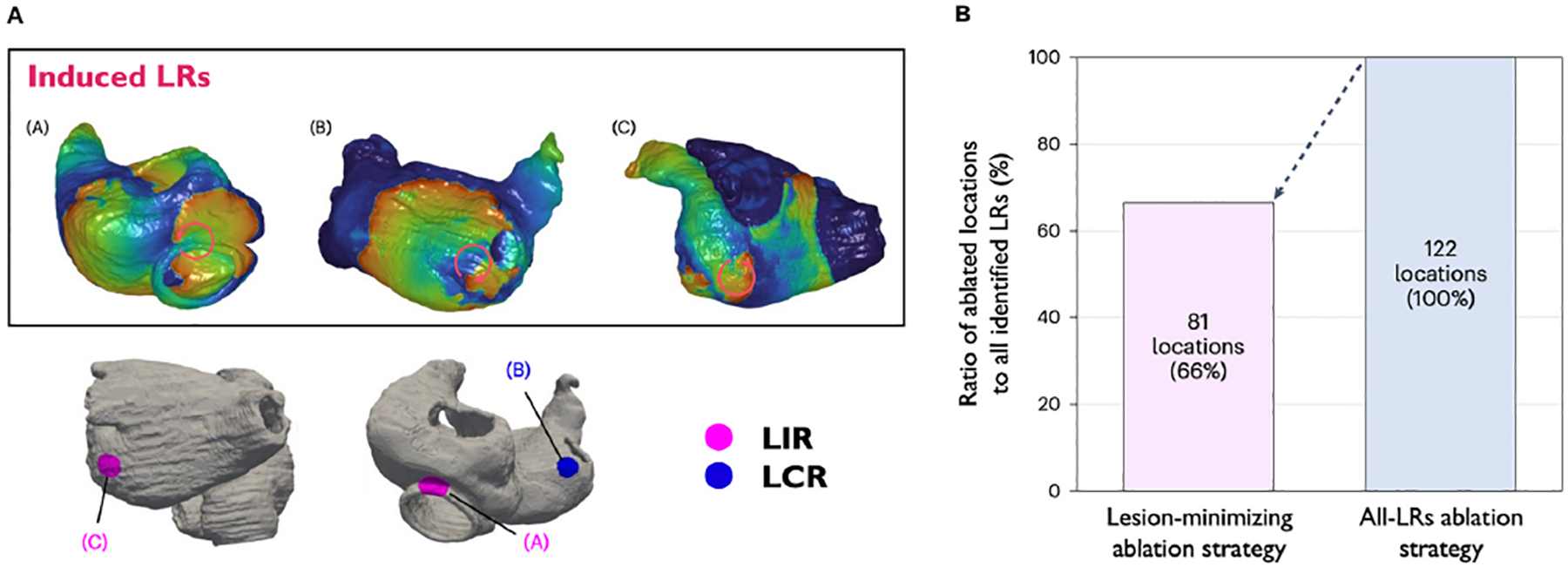
Discernment of contingent LRs from identified LRs in the DT A. (top) Three LRs were identified in the DT. (bottom) Among 3 LRs, two were LIRs that required ablation to achieve arrhythmia non-inducibility and one was an LCR that lost its rotor-attracting capabilities when another LR was eliminated. B. By discerning LCRs from all identified LRs, this new “selective” approach to substrate ablation reduced the number of target locations by 34% over the entire DT patient cohort, thereby mitigating unnecessary extensive ablation and heart damage. DT = digital twin; LCR = location of contingent reentry; LIR = location of reentry; LR = location of reentry. Adapted from Sakata et al., *Nature Cardiovascular Research*, 2024.

To mitigate unnecessary heart damage while controlling PsAF arrhythmogenesis, personalized DTs revealed that among all LRs uncovered by sequential pacing from a large number of bi-atrial sites, there is a group of LRs, termed “contingent LRs” that disappear when a different site in the atria is ablated. Thus, contingent LRs do not need to be ablated to achieve arrhythmia non-inducibility and therefore should not be considered therapeutic targets, thereby establishing selective ablation strategy (Sakata, Bradley, Prakosa, Yamamoto, Ali, et al., 2024). Fig. 5A shows an example case of PsAF patient. In this patient DT, three LRs were identified post-virtual PVI and then ablating them rendered LRs non-inducible (all-LRs strategy). However, by sequentially ablating each LR one by one, one LR was identified as a contingent LR and ablating only the other two LRs achieved non-inducibility (lesion-minimizing strategy). Overall, among 122 identified LRs, lesion-minimizing strategy reduced the number of ablation targets by 34% (Fig. 5B).

**Fig. 6.**
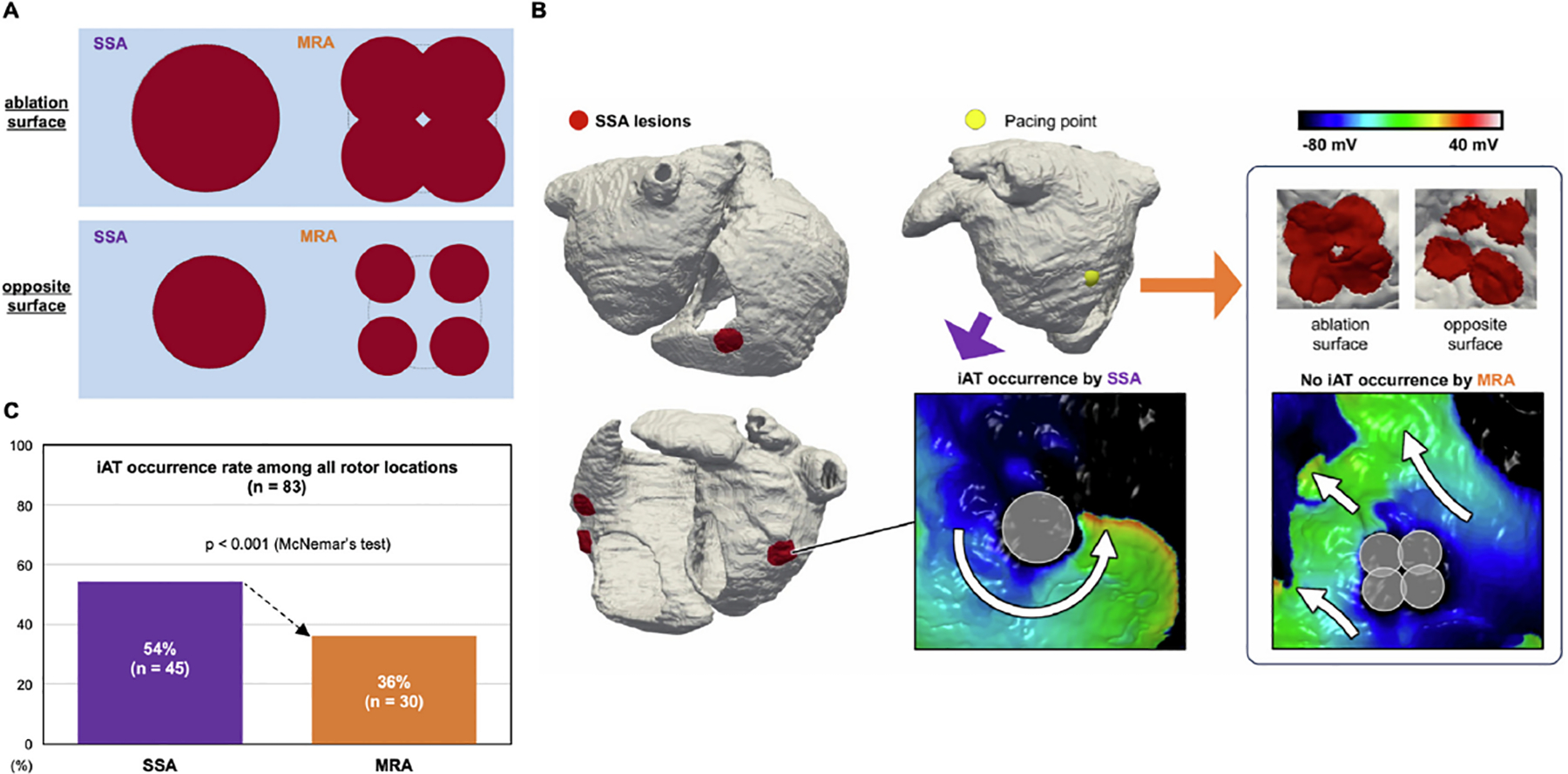
Preventing scar-related macroreentrant AT by MRA A. (top) SSA lesions mimicked contiguous overlapping lesions created by radiofrequency ablation with normal power, forming a transmural compound lesion. (bottom) MRA lesions consisted of 4 smaller lesions resulting in the lesions on the opposite atrial surface not being connected to each other, avoiding a large anatomic obstacle favorable to reentry formation. Light-blue area indicates the atrial wall and red area indicates ablated scar tissue. B. SSA lesion executed at an LR caused scar-related macroreentrant AT, i.e., iAT, around the SSA lesion (left), while MRA lesion executed at that LR resulted in the prevention of iAT (right). C. Compared with SSA, MRA significantly reduced the occurrence rate of iAT by 18% across all LRs identified in the patients’ DTs. AT = atrial tachycardia; DT = digital twin; iAT = post-ablation iatrogenic AT; LR = location of reentry; MRA = multiple reduced-strength ablation; SSA = single-region standard ablation. Adapted with permission from Sakata et al., *JACC. Clinical electrophysiology*, 2024.

In a different study, personalized DTs further showed that, even with similar transmural lesion creation, scar-related reentry formation can be avoided depending on how ablation is delivered (Sakata, Bradley, Prakosa, Yamamoto, Yusuf Ali, et al., 2024). By avoiding a large anatomical obstacle on the opposite atrial surface to ablation application—favorable to reentry formation (Fig. 6A), such ablation lesions enabled propagating wavefronts to take alternative pathways and escape without forming scar-related reentry (Fig. 6B). Overall, among 83 LRs in the entire cohort, this new ablation approach significantly reduced the occurrence rate of scar-related reentry by 18% (Fig. 6C).

**Fig. 7.**
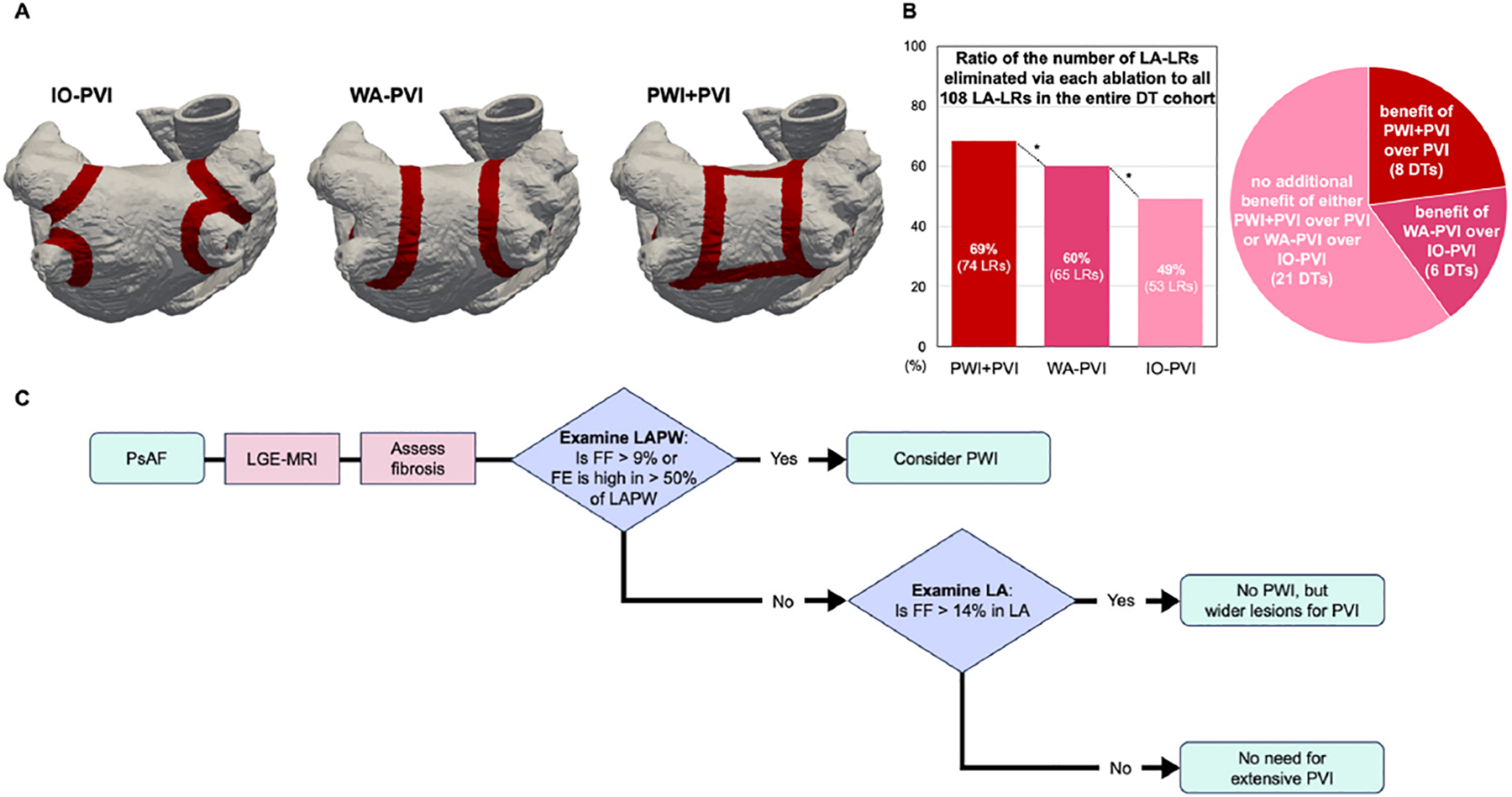
Patient stratification benefitting from WA-PVI or PWI + PVI A. (left) IO-PVI. (bottom) WA-PVI. (right) PWI + PVI. Red lines indicate linear ablation lesions. B. (left) PWI addition eliminated more LA-LRs than WA-PVI (69% vs. 60%) in the DT cohort, and WA-PVI was also significantly more beneficial than IO-PVI (60% vs. 49%) in diminishing LA substrate arrhythmogenicity. (right) In 60% of all PsAF patients’ DTs, PVI greatly decreased LA substrate arrhythmogenicity without the need of wider lesions or additional PWI. C. Fibrosis assessment could be useful for properly stratifying patients benefitting from PWI or wider PVI. DT = digital twin; FE = fibrosis entropy; FF = fibrosis fraction; IO-PVI = individual ostial PVI; LA = left atrium; LAPW = LA posterior wall; LGE-MRI = late gadolinium enhancement magnetic resonance imaging; LR = location of reentry; PsAF = persistent atrial fibrillation; PVI = pulmonary vein isolation; PWI = LAPW isolation; PWI + PVI = LAPW isolation plus WA-PVI; WA-PVI = wide antral PVI. Adapted from Sakata et al., *NPJ digital medicine*, 2025.

Furthermore, DTs have demonstrated that PVI, primarily designed for trigger isolation, has a considerable impact on LR elimination (Sakata et al., 2025). To investigate their ablation impacts on LA substrates, three ablation strategies, two different PVI configurations—individual ostial PVI (IO-PVI) and wide antral PVI (WA-PVI)—and LA posterior wall isolation (PWI) plus PVI, were compared (Fig. 7A). PWI addition eliminated more LA-LRs than WA-PVI (69% vs. 60%) in the DT cohort, and WA-PVI was also significantly more beneficial than IO-PVI (60% vs. 49%) in diminishing LA substrate arrhythmogenicity; however, in 60% of all PsAF patients’ DTs, PVI greatly decreased LA substrate arrhythmogenicity without the need of wider lesions or additional PWI (Fig. 7B). Fibrosis assessment could be useful for properly stratifying patients benefitting from PWI or wider PVI (Fig. 7C). Similarly, DTs revealed that in cases of sustained AF despite complete PVI, comparable attention to the RA to the LA may be necessary for PsAF substrate management (Sakata et al., 2026).

Similarly, patient-specific atrial DT-based simulations enabled iterative identification of high dominant frequency regions sustaining AF and generation of minimal and personalized ablation lesion sets and lines, achieving >98% simulated AF termination while ablating only 5–6% of the atrial myocardium (Azzolin et al., 2023). These findings demonstrated that patient-specific DTs can be used to design personalized ablation strategies capable of terminating AF based on underlying arrhythmogenic substrate, highlighting that effective AF termination requires accurately identifying and ablating patient-specific drivers within DTs, rather than structural fibrosis or electrophysiological abnormalities themselves, in isolation.

To systematically evaluate the efficacy of personalized ablation strategies, recent studies have reported that DT-based simulations enable systematic comparison of alternative ablation strategies and their patient-specific efficacy. Personalized atrial DTs were used to simulate and compare multiple anatomical, structural, and functional ablation strategies, demonstrating that optimal lesion sets vary across patients (Roney et al., 2020). Extending this approach to cohort-level analysis, a virtual cohort of 1000 atrial DTs derived from statistical shape models was employed to systematically simulate different treatment strategies and predict ablation outcomes using multimodal structural and electrophysiological feature maps, demonstrating that fibrosis-guided ablation strategies significantly improved simulated AF termination (Zolotarev et al., 2025). These findings demonstrated that DT-based simulations enable not only personalized ablation strategy design but also systematic evaluation and rapid prediction of their therapeutic efficacy without explicitly simulating each ablation strategy.

Recently, DT-based approaches that enabled automated identification of ablation targets and lesion design beyond manual identification of PsAF arrhythmogenesis have been reported. Personalized atrial DTs enabled automatic identification of extra-PVI ablation targets and generation of personalized lesion masks using electro-optic flow mapping, reducing simulated AF inducibility while minimizing ablated tissue (Jaffery et al., 2026). These findings demonstrated that DT-based simulations can automate personalized ablation planning, thereby facilitating scalable and objective personalization of ablation strategies.

Beyond optimal target identification and ablation strategies, patient-specific DTs also enabled prediction of individual pharmacological response after AF ablation. By integrating clinical electroanatomic mapping and computed tomography-derived atrial geometry, DTs simulated the electrophysiological effects of virtual amiodarone administration and classified patients based on virtual AF termination (Hwang et al., 2024). In this study, patients whose DTs exhibited AF termination in response to virtual antiarrhythmic drug testing showed significantly lower clinical recurrence during 1-year post-ablation, demonstrating that DTs can guide personalized pharmacological therapy selection.

Finally, recent studies have reported advances in model personalization and electrophysiological calibration, highlighting the ongoing evolution of DT technology and its increasing potential to enable precise, personalized ablation strategies and facilitate its smooth clinical adoption, such as the construction of personalized heart DTs using non-invasive ECGI data to allow rapid calibration of electrophysiological parameters and accurate reproduction of patient-specific activation patterns, with potential applications in ablation planning (Herrero-Martín et al., 2025).

Collectively, these studies demonstrate the potential of personalized DTs to enable personalized ablation planning, predict treatment outcomes, and optimize post-ablation management in patients with AF.

## 6. Conclusions and outlook

In this review, we summarized the arrhythmogenic substrate mechanisms of PsAF, the historical development of substrate ablation strategies, current clinical limitations of substrate ablation, and the emerging role of DTs in optimal ablation strategies. The rapid advancement of DT technology has the potential to embody the concept of precision cardiology. However, atrial DTs do not fully reproduce all aspects of physiological reality in the clinical setting. Indeed, most DT studies still rely on simplified or incompletely calibrated parameters, because a central priority has often been to use clinical data that can be obtained non-invasively. This is partly attributable to the limited capabilities of current modeling technologies including calibration and computational simulation technologies (Zappon et al., 2026). Moreover, other complex regulatory mechanisms are not yet fully integrated, including patient-specific autonomic nervous system activation, which can facilitate reentries (Vandenberk et al., 2024). Future developments of computational frameworks may further improve the representation of inter-patient variability, including differences related to sex, age, and comorbidities. In addition, practical barriers remain for large-scale clinical validation of atrial DT-guided ablation. These includes the technical difficulty of acquiring high-quality atrial LGE-MRI for reliable fibrosis characterization, the limited integration of such imaging into routine AF ablation workflows and clinical guideline, and the substantial expertise and computational resources required for DT construction and electrophysiological simulations. Nonetheless, the limitations of ad-hoc ablation strategies have become evident over the past decades. In this context, the use of non-invasive, mechanism-based insights obtained in the pre-procedural setting is expected to become increasingly important.

## Figures and Tables

**Table 1:** Representative strategies for PsAF substrate ablation.

	Representative clinical studies
CFAE	Nademanee et al., 2004; Verma et al., 2015; Ohe et al., 2019
Stepwise	Haïssaguerre et al., 2005; Vogler et al., 2015; Inoue et al., 2021
DF	Verma et al., 2011; Kumagai et al., 2013; Atienza et al., 2014
GP	Nakagawa et al., 2009; Pokushalov et al., 2013; Berger et al., 2019
LVA	Rolf et al., 2014; Huo et al., 2022; Masuda et al., 2025
LGE-MRI-based fibrosis	Marrouche et al., 2014; Marrouche et al., 2022
FIRM	Narayan et al., 2012; Kirzner et al., 2019; Brachmann et al., 2025
ECGI	Haissaguerre et al., 2014; Knecht et al., 2017; Honarbakhsh et al., 2022
CARTOFINDER	Calvo et al., 2017; Takahashi et al., 2020; Chang et al., 2022
STED	Seitz et al., 2017; Qin et al., 2020; Deisenhofer et al., 2025
ExTRa Mapping	Sakata et al., 2018; Kawaji et al., 2025; Okano et al., 2025

CFAE = complex fractionated atrial electrogram; DF = dominant frequency; ECGI = electrocardiographic imaging; FIRM=Focal Impulse and Rotor Modulation; GP = ganglionated plexi; LGE-MRI = late gadolinium enhancement magnetic resonance imaging; LVA = low-voltage area; PsAF = persistent atrial fibrillation; STED = spatiotemporal electrogram dispersion.
